# Psychological experiences of women with pregnancy termination due to fetal anomalies: a qualitative study from the perspective of women, their spouses, and healthcare providers in Iran

**DOI:** 10.1186/s12978-020-00959-y

**Published:** 2020-07-09

**Authors:** Bahareh Kamranpour, Mahnaz Noroozi, Masoud Bahrami

**Affiliations:** 1 Department of Midwifery, College of Nursing and Midwifery, Rasht Branch, Islamic Azad University, Rasht, Iran; 2 Department of Midwifery and Reproductive Health, School of Nursing and Midwifery, Isfahan University of Medical Sciences, Isfahan, Iran; 3 Department of Adult Health Nursing, School of Nursing and Midwifery, Isfahan University of Medical Sciences, Isfahan, Iran

**Keywords:** Psychological needs, Pregnancy termination, Fetal anomalies, Care, Qualitative study

## Abstract

**Background:**

Pregnancy termination due to fetal anomalies has many psychological consequences for women. Providing appropriate and desirable care to this group of women and their families plays an important role in the process of coping with this crisis. The aim of the present study was to explore the psychological experiences of women with pregnancy termination due to fetal anomalies.

**Methods:**

This was a qualitative content analysis study. 40 participants were selected through purposeful sampling with maximum variation and data were collected through in-depth individual interviews, field notes, and analyzed using the conventional qualitative content analysis method simultaneously.

**Results:**

After analyzing the interview transcripts, the psychological experiences of women with pregnancy termination due to fetal anomalies were classified into two main categories: “emotional reactions coinciding with the diagnosis of fetal anomalies” (consisting of two sub-categories of “disbelief and denial of fetal anomalies” and “feelings of sadness and anger”) and “ psychological problems following pregnancy termination” (consisting of two sub-categories of “ feeling helpless, fearful, anxious, and depressed” and “feeling conscience-stricken, and guilty”).

**Conclusion:**

According to findings of the present study, exploring and highlighting the experiences of women with pregnancy termination due to fetal anomalies in the psychological dimension can provide a deeper understanding of the needs of these women for providing optimal care at different times and ultimately promote their psychological health.

## Plain English summary

Diagnosis of fetal anomalies is an unexpected event and causes severe emotional harm to women. Although women still does not have a tangible memory of her child in these cases, the severity of the grief that results from this loss is no different from other causes of fetal loss such as abortion and intrauterine fetal death. Approximately all studies have reported evidence of these reactions, especially in the first few weeks and months after pregnancy termination. The present qualitative study was conducted to explore the psychological experiences of women with pregnancy termination due to fetal anomalies. In this study a total of 40 participants, including women with pregnancy termination due to fetal anomalies, their spouses and healthcare providers in Isfahan, Iran were selected and interviewed. Psychological experiences of these women were: emotional reactions coinciding with the diagnosis of fetal anomalies (including “disbelief and denial of fetal anomalies” and “feelings of sadness and anger”) and psychological problems following pregnancy termination (including “feeling helpless, fearful, anxious, and depressed” and “feeling conscience-stricken and guilty”). In conclusion; exploring and highlighting the psychological experiences of these women and presenting them to those involved in the field of psychological health, can provide a deeper understanding of how to policy and provide optimal care at different times for them and improve their psychological health.

## Background

Broad advances in diagnostic technologies and the widespread and early application of imaging techniques have led to early diagnosis of pregnancy and maternal fetal attachment. Considering the decline in fetal mortality rates, mothers have higher expectations of successful pregnancy outcome [1].

Diagnosis of fetal anomalies is an unexpected event and causes severe emotional harm to women [2]. Although parents still do not have a tangible memory of their child in these cases, the severity of the grief that results from this loss is no different from other causes of fetal loss such as abortion and intrauterine fetal death (IUFD) [3]. On the other hand, the necessity to deciding on the fate of the fetus complicates the grief and has many psychological consequences [4]. Loss of self-esteem due to a feeling of physical inadequacy in giving birth to a healthy child, assuming moral responsibility for deliberate fetal death, and the stress due to fear of social judgment lead to greater loneliness and vulnerability in these women [5].

Studies showed that women with pregnancy termination due to fetal anomalies do not receive adequate support, and even cannot mourn the death of their child due to lack of public awareness of this type of loss [6]. Even if the decision to terminate pregnancy is the best choice for the current situation, it will have many psychological consequences for the parents. Approximately all studies have reported evidence of these reactions, especially in the first few weeks and months after pregnancy termination [7]. The level of psychological consequences may even don’t decrease in the first few years after the event, as the results of the study by Maguire et al. showed that 17% of women experienced symptoms of post-traumatic stress for 2 to 7 years after pregnancy termination due to fetal anomalies [8]. Studies revealed that majority of these women have symptoms of depression, sleep disturbances, restlessness, nutritional problems, and approximately 14% of them suffer from symptoms of severe depression [4]. Even mild psychological reactions during this critical period are a very important predictor of long-term adverse psychological consequences [9]. There are currently no specific support systems for this group of women. Although there are currently online groups in some countries to support women experiencing pregnancy termination due to fetal anomalies, it is not yet clear whether this form of support fully fits the needs and desires of this population [10].

Qualitative research is an approach to discovering and describing people’s experiences and conceptualizing them which leads to increased insight, understanding and awareness about human experiences [11]. Considering the different dimensions of the experience of pregnancy termination due to fetal anomalies [12], conducting a study, particularly a qualitative study can provide a deeper understanding of the psychological experiences of these women in their social-cultural context. Understanding these psychological experiences can be used in designing and implementing care based on the needs and desires of these women. Therefore, the present qualitative study was conducted to explore the psychological experiences of women with pregnancy termination due to fetal anomalies.

## Methods

### Study design

The present qualitative research used a content analysis approach and conducted between October 2017 and April 2018.

### Settings, sample and recruitment

Participants included 25 women with pregnancy termination due to fetal anomalies referred to hospitals in Rasht, Iran, two of their spouses and 13 healthcare providers (two forensic medicine specialists, three gynecologists, one perinatologist, one psychologist, two reproductive health specialists, three midwives and one nurse) who were selected through purposeful sampling. Then, sampling was continued with maximum variation (in terms of age, educational level, occupation, number of children and time elapsed since pregnancy termination). Participants were accessed through the midwifery wards of the hospitals, prenatal clinics, and midwifery, gynecologists and forensic medicine specialists’ offices. They were recruited directly or telephone numbers were obtained and they were subsequently telephoned. Inclusion criteria included participants’ willingness to participate in the study and informed consent, the ability to understand questions and having reading and writing literacy, a maximum of 1 year elapsed since pregnancy termination, and the absence of any known psychological illness. After reaching eligible participants, none of them refused to participate in the study.

### Data collection

Data were collected through semi-structured in-depth face-to-face interviews, and field notes. The first author (BK), who has 12 years working experience in midwifery, conducted the interviews and field notes. Other authors have previous interviewing experience and qualitative paper/report writing. Prior to data collection, the first author wrote down initial preconceptions and beliefs about the research topic based on her previous working experience and from a review of the literature. Interview began with women using general questions including “Please explain your feelings when you informed about your fetal abnormality and you decided to terminate the pregnancy? and “Please explain what emotional problems did you experience when you became aware of your fetal abnormality and that pregnancy should be terminated?“ and then the participants’ open-ended and detailed responses guided the process. Interview began with spouses of participating women using a general question “Please explain what your spouse emotional problems were when she became aware of the fetal abnormality and that her pregnancy should be terminated?” Also, interviews began with other participants (healthcare providers) using a general question “Based on your experiences, what are the psychological experiences of women with pregnancy termination due to fetal anomalies?” In this study, forty 25–100 min interviews were conducted in locations chosen by the participants. Interviews continued until reaching data saturation. The demographic characteristics of the participants are presented in Tables 1, 2.

**Table 1:** Demographic characteristics of women with pregnancy termination due to fetal anomalies and their spouses

Characteristic	Number
**Age (years)**	
20–30	15
31–40	12
Total	27
**Level of education**	
Middle school	3
Diploma	8
Associate degree	2
BS	12
MS and Ph.D	2
Total	27
**Job**	
Employee	9
Housewife	7
Service	10
Free	1
Total	27
**Number of children**	
No children	14
1 to 2 children	13
Total	27
**Time elapsed since pregnancy termination**	
Less than 6 months	24
6 months to one year	3
Total	27

**Table 2:** Demographic characteristics of other participants (healthcare providers)

Characteristic	Number
**Age (years)**	30–60 **(**13)
**Work experience (years)**	1–30 **(**13)
**Occupation**	Gynecologist (3), Perinatologist (1), Forensic Medicine Specialist (2), Reproductive Health Specialist (2), Midwife (3), Nurse (1), Psychologist (1)

### Data analysis

Data analysis was carried out using conventional qualitative content analysis [11]. In this study, any software was used to analyze the data. The first author (BK) transcribed the data on a regular basis as each interview was conducted and recorded using an mp4 player. The interviews were then repeatedly reviewed to obtain a complete understanding of them. The sentences and phrases were then coded, and after the codes were formed in an inductive manner, the same codes were merged and those that had a similar concept were grouped together and formed into sub-categories. Subsequently, the sub-categories were compared using the inductive method, and similar sub-categories were put into the same main category.

### Rigor and trustworthiness

In order to ensure trustworthiness of the obtained content, coded interviews were consulted with four participants in other sessions and their final comments were summarized in order to allow participants to review the interviews. Different methods were used to ensure trustworthiness of the findings, including in-depth interviews at different times and locations, combining several data collection methods such as individual interviews and field notes, and selecting the different groups of participants (women, their spouse and healthcare providers) with maximum variation. In the present study, in order to increase transferability, the findings of the study were presented to three women with similar characteristics who experienced pregnancy termination due to fetal anomalies and did not participate in the study, to judge regarding the similarity of the results of the study with their experiences. To ensure that the findings were consistent with the participants’ statements, the opinions of four experts were also used.

### Ethical considerations

The research approval of the Research Ethics Committee of Vice Chancellor for Research of Isfahan University of Medical Sciences (ethical code: IR.MUI.REC. 1395.3.945) was obtained and informed consent, anonymity, confidentiality of information and right of withdrawal at any time were also taken into account.

## Results

After analyzing the data, 31 codes, four sub-categories and two main categories were obtained. The two main categories include “emotional reactions coinciding with the diagnosis of fetal anomalies “and “psychological problems following pregnancy termination” (Table 3).

**Table 3:** Codes, sub-categories and main categories extracted from the data analysis

Code	Sub-category	Main Category
-Unexpected diagnosis of fetal anomalies	Disbelief and denial of fetal anomalies	Emotional reactions coinciding with the diagnosis of fetal anomalies
-Difficult nature of accepting reality		
-Unacceptable nature of the reality of fetal anomalies		
-Being sad to hear about fetal anomalies	Feeling sad and angry	
-Crying a lot		
-Getting angry to hear about fetal anomalies		
-Losing control		
-Feeling unable to resolve the problem	Feeling hopeless, afraid, anxious and depressed	Psychological problems following termination of pregnancy
-Feeling unable to give birth to a healthy child		
-Feeling afraid of the re-occurrence of fetal anomalies		
-Feeling fearful of relative’ misjudgments		
-Feeling unwell and bored		
-Feeling apprehension and anxious		
-Feeling restless and disinterested in doing everyday tasks		
-Feeling the conscience-stricken about being involved in an fetal abnormality	Feeling guilty	
-Feeling guilty about the fetal anomalies		
-Feeling guilty about interfering with the pregnancy termination and killing the fetus		

### Emotional reactions coinciding with the diagnosis of fetal anomalies

According to the healthcare providers, since women and their spouses knew of fetal anomalies suddenly and unexpectedly while they were expecting a healthy child, loss of the fetus can be a major emotional blow to them under these conditions. Approximately, all participating women had experienced a critical period of emotional and psychological problems as the fetal abnormality was diagnosed. This main category consists of two sub-categories.

#### Disbelief and denial of fetal anomalies

Participating women as well as their spouses narrated that the major psychological shock for women was the diagnosis of fetal anomalies, causing many psychological reactions. One of the worst reactions was the rejection of the reality of fetal anomalies. This fact was so painful for women that they initially denied it.

*" … When the ultrasound revealed that my fetus had a brain problem, I didn't accept it and didn't suffice to this ultrasound. I underwent sonography, but the result was the same. It was a surprise to me because there was no previous history of such problem. But, the doctors said this fetus was going to die after a while and I had to abort it, but again, I was not satisfied, saying that the ultrasound might be wrong. “(Woman)*

*“ … Our two previous children are healthy. When we were told that this fetus had an abnormality, it was very difficult for my wife to accept it.”(Husband)*

Also, healthcare providers believed that one of the most important concerns for pregnant women is ensuring the health of the fetus. Therefore, it is very painful for them to know that the fetus is abnormal.

*“When pregnancy occurs, women have only one thing to think about is giving birth to a healthy baby. All screening tests are a means of assuring them that the fetus is healthy. For example, they see the ultrasound appointment as an opportunity to see the fetus or to know her/his gender and don’t expect to hear anything else at all.” (Reproductive health specialist)*

#### Feelings of sadness and anger

Participating women narrated that being aware of fetal anomalies is regarded as death of a child and the loss leads to a serious and big sadness to the extent that they start mourning. Most of the participating women showed responses such as anger, sadness, and crying with varying degrees in response to the unexpected loss of the fetus.

*" … You just enjoy having a baby ... you're getting accustomed to the baby ... every family member knows about it... you think about his/her name ... his/her stuff ... whether he's a boy or a girl ... that you suddenly find out that the fetus is abnormal and you have abort it ... that means you have no choice ... it really makes you feel bad.”(Woman)*

Also, participating men believed that their spouses have experienced a critical period of psychological problems coinciding with the diagnosis of fetal anomalies and needed to alleviate these problems.

*"After hearing that our fetus was abnormal, my wife cried a lot!!! She tried to control herself but could not. "(Husband)*

### Psychological problems following pregnancy termination

According to participants’ statements, women experience unpleasant emotions such as disability, fear, anxiety, and depressed mood after termination of pregnancy. Also, in these women the misconception that being involved in the fetal anomaly leads to a sense of guilty. This main category consists of two sub-categories.

#### Feeling helpless, fearful, anxious, and depressed

According to the participating women, their inability to resolve the problem made them feel completely helpless. Also, the feeling of helplessness and frustration in preventing the reoccurrence of the same incidence made them feel that they were unable to bear a healthy child in the future. A number of participating women believed that the treatment team or even pre-conception counseling could not be effective in preventing the reoccurrence of the incidence.

*"It was the first time in my life that I had a problem that I could not do anything ..., so to speak, incurable pain... Despite the advances in medical science, there is no way to save the baby." (Woman)*

The majority of participating women narrated that they have experienced unpleasant emotions such as fear and anxiety about recurrence of the incidence, fear of misjudgment about the fetus and the cause of the incidence by the relatives, anxiety and depression after pregnancy termination.

*"I didn’t get out of the house for a week when I was discharged. It was hard for me. I didn't know what to do. What should I if it happens again? My anxiety never goes away." (Woman)*

According to a number of participating women, the unpleasant feeling of depression was so serious that it made them unable to perform the daily tasks of their lives.

*"I was depressed for a month after the abortion, and I felt very bad as I felt thunderstruck for a moment. I didn’t really feel like doing anything. I was bored. I wanted to sleep all the time." (Woman)*

#### Feeling conscience-stricken, and guilty

Participating women narrated that they experienced feelings of guilt over their involvement in the occurrence of fetal anomalies and the decision to terminate their pregnancy.

*"I have no idea what was the cause of it (fetal anomalies). There was no history of this in my family and that of my husband. Maybe, when I doubted that I was pregnant and brew saffron and drank it, maybe I was effective, now I feel guilty and say that I may made this trouble for myself.” (Woman)*

Healthcare providers believed that if women were received complete, adequate, and appropriate information about the possible causes of fetal anomalies by the medical practitioner and other medical staff; they would not have experienced this confusion and feeling of guilt.

*“ … Parents should be informed about possible causes of fetal anomalies. This should be done in a way that they don’t feel guilty.” (Psychologist)*

## Discussion

The aim of the present study was to explore the psychological experiences of women with pregnancy termination due to fetal anomalies. The results of this study showed that emotional reactions coinciding with the diagnosis of fetal anomalies and psychological problems following pregnancy termination are among the most important psychological experiences of these women.

According to the participants, women were shocked and had disbelief after being informed of the fetal anomalies. In this regard, the results of the study by Eklein et al. showed that most parents were unprepared to be diagnosed with fetal anomalies, and faced high levels of psychological distress. In addition, while responding to this crisis, they find that they were wrong to have a sense of security they experienced because of the advances in medical sciences [13]. This highlights the importance of receiving psychological support in helping to accept reality and coping with this crisis.

The results of the present study showed that women experienced grief and sorrow when they became aware of fetal anomalies and the need to have an abortion. Results of Asplin’s study also revealed that most women experienced a range of unpleasant emotions such as grief, futility, loneliness, fatigue, and anger. However, experienced caregivers can play an importance role in affirming their feelings, showing friendliness, and dignity in speaking and behaving [14]. So, according to the results of the present study, important factor in coping with this crisis seem to be the presence of professional caregivers who provide ongoing care and appropriate psychological support to women based on their psychological needs.

The results of the present study showed that women experienced fear, anxiety, and depression after pregnancy termination. Similarly, the results of Kong’s study showed that most patients believed that the psychological experience due to loss of the fetus could have a serious impact on women’s health, and that psychological support should be provided after the loss of the fetus [15].. Also, in the present study, most participants expressed a feeling of helplessness to confront and accept reality, failure to solve the problem, and inability to give birth to a healthy child. The results of Asplin’s study showed that most women had long been in doubt after pregnancy termination, and did not know whether they could have a healthy child in the future [16]. Therefore, it seems that providing psychosocial support by professional caregivers is valuable and can lead to fewer psychological problems.

Many of the participants in the present study were afraid and anxious about the misjudgment of relatives regarding the fetal anomalies and the cause of the incidence. The results of McCoyd’s study also show that stigma of abortion and having an abnormal fetus makes the women fearful of being judged by relatives, and couldn’t share their grief with them, and thus didn’t receive sufficient support under these situations [17]. So, healthcare providers should be more sensitive to the adverse mood and emotional consequences of fetal loss as well as the unmet needs of these individuals.

The results of the present study indicated that some of the participants had feeling severe depression. These people stated that they were unable to perform the daily tasks of life and even felt that their lives were worthless. Similarly, the results of various studies have shown that the sense of responsibility to make decisions regarding termination of pregnancy leads to an increase in the severity of grief following pregnancy termination in women [18, 19]. So, emotions such as unwillingness to continue living should make caregivers sensitive to the special needs of these women and they should follow up and take the necessary measures in this regard.

The results of the present study showed that a misconception about the possible cause of fetal anomalies has led to the feeling of guilt about fetal anomalies and feeling conscience-stricken about ending the fetal life in most participants. In this regard, most participants in Maguire’s study referred to self-blaming for the decision to terminate pregnancy, social isolation, and the grief associated with remembering memories [8]. Therefore, in addition to providing counseling services, evaluating women’s psychological health status, conducting psychotherapy sessions, taking into account the impacts of such loss on women and their families, and focusing on future problems facing these women will increase their understanding of their new situation and significantly help them adjust to the existing conditions [20, 21].

Through presenting an image of psychological experiences of women with pregnancy termination due to fetal anomalies, for the first time, the present study is able to help for designing the necessary interventions to provide optimal care at different times for these women and improve their psychological health. The present study did not include women who were experiencing pregnancy termination due to fetal anomalies for more than 1 year. Since a number of studies have shown that associated consequences will be persist for more than 1 year [11, 22], restricting samples to a maximum of 1 year after pregnancy termination can be one of the limitations of the present study.

## Conclusion

This study revealed that women with pregnancy termination due to fetal anomalies experienced emotional reactions coinciding with the diagnosis of fetal anomalies (including disbelief and denial of fetal anomalies, feelings of sadness and anger) and psychological problems following pregnancy termination (including feeling helpless, fearful, anxious, and depressed and feeling conscience-stricken, and guilty). It seems that exploring and highlighting the psychological experiences of these women can be used to provide the optimal care at different times for them and ultimately promote their psychological health.

## Data Availability

The datasets generated and/or analysed during the current research are not publicly available as individual privacy could be compromised but are available from the corresponding author on reasonable request.

## Abbreviation

IUFD: Intra Uterine Fetal Death
